# Periovulatory Hormonal Profiles after Estrus Induction and Conception Rate by Fixed-Time AI in Payoya Goats during the Anestrous Season

**DOI:** 10.3390/ani12202853

**Published:** 2022-10-20

**Authors:** Francisco A. Arrebola, Rafael Torres-Martell, Olga González-Casquet, Cesar A. Meza-Herrera, Carlos C. Pérez-Marín

**Affiliations:** 1 Instituto de Investigación y Formación Agraria y Pesquera (IFAPA), Carretera el Viso, km 2, 14270 Hinojosa del Duque, Spain; 2 Diputación de Cádiz, 11400 Jerez de la Frontera, Spain; 3 Asociación Nacional Criadores Cabra Payoya, 11680 Algodonales, Spain; 4 Unidad Regional Universitaria de Zonas Áridas, Universidad Autónoma Chapingo, Texcoco 35230, Mexico; 5 Department of Animal Medicine and Surgery, Faculty of Veterinary Medicine, University of Córdoba, 14014 Córdoba, Spain

**Keywords:** hormone, insemination, Payoya goat, sponge, estrus induction

## Abstract

**Simple Summary:**

Standardized induction and synchronization treatments and AI timing are used in goats, despite the fact that physiological differences can be linked to the breed, which could compromise the achievement of the AI programs in autochthonous breeds. However, few, if any, studies have been carried out to explore the reproductive physiology in the autochthonous breeds, in contrast to the commercial breeds. We studied the periovulatory hormone-release in Payoya goats, an autochthonous, endangered Spanish breed, and some differences were detected between the pregnant and non-pregnant goats. We observed a lower LH peak in those goats that failed to get pregnant, whilst other periovulatory hormones showed a similar release pattern in both groups. The results suggest that the LH peak should be favored to increase the success of fixed-time artificial insemination programs, and further studies should be conducted to understand the cause of this diminished LH release during the preovulatory surge.

**Abstract:**

Sexual activity in domestic goats is positively influenced by reducing the photoperiod. Various protocols have therefore been developed in goats for the induction and synchronization of estrus during those months in which their sexual activity is reduced. The present observational study evaluates the periovulatory hormonal profile in Payoya goats (n = 24), during a non-favorable photoperiod (i.e., spring), being treated for estrus induction. The treatment comprised the vaginal insertion of sponges impregnated with progestogen (fluorogestone acetate, FGA), together with cloprostenol and equine chorionic gonadotrophin (eCG), 48 h before the end of the treatment. When the treatment ended, the plasma concentrations of the LH, FSH, progesterone and estradiol were determined. The goats were inseminated 46 h after the sponge withdrawal, and a pregnancy diagnosis was carried out 40–45 days after the insemination. Various parameters were monitored, such as the peaks of luteinizing hormone (LH), follicle-stimulating hormone (FSH) and estradiol, and their respective intervals, in reference to the time of the sponge withdrawal. The conception rate was 62.5%, and the kidding rate was 50%. The results record the hormonal release pattern after the estrus synchronization treatment based on the FGA, and the differences between the pregnant and non-pregnant goats. The findings suggest that the LH peak produced after the estrus synchronization treatment, both in terms of the amplitude and the time of increment, is involved in the reproductive failure detected.

## 1. Introduction

Goats exhibit a seasonal reproductive pattern under the influence of photoperiodicity, which determines their annual reproductive cycles. This physiological characteristic constitutes a drawback in terms of producing milk and meat, given that producers aim to maintain constant output levels over the course of the year [1,2]. Estrus induction and synchronization treatments are frequently used in goats as a tool to overcome the non-breeding periods, favoring the hormonal production needed to reach ovulation and obtain an optimum reproductive performance. When artificial insemination is implemented, estrus induction and synchronization treatments must be used. Progestogen-based treatments are habitually used following standard protocols, but physiological differences can be associated with the breed. It is therefore desirable to know the specific hormone release pattern for each breed to design more efficient treatments. This is particularly important when treatments are used in local breeds, where there is little in the scientific literature about their reproductive physiology, and the implementation of AI has significant potential.

In order to implement appropriate measures for controlling estrus in goats, it is useful to establish the reproductive peculiarities of the different breeds. Hormonal treatments for estrus induction and synchronization are a key tool in obtaining good reproductive results using artificial insemination in goats, and they could help to improve output and farm incomes as a consequence [2].

It has been reported that the interestrus interval during the breeding season in goats is around 20–21 days, being more regular at the beginning of the season. The mean duration of the estrus signs is 36 h, with a range of 24 to 48 h. Ovulation occurs 20–26 h after the LH peak, which usually takes place 12 h after the first signs of heat [3].

During the follicular phase, high GnRH levels are produced from the hypothalamus, stimulating the pituitary gland. The released FSH then promotes the follicular growth and initiates a new follicular wave, which leads to the selection of 2–3 dominant follicles. The pituitary gland also releases LH, which strengthens the development of the preovulatory follicles (reaching around 6–9 mm), while the rest of the cohort is atretic. Just before the beginning of the heat signs, 17-β-estradiol is released from the preovulatory follicles, reaching around 20–50 pg/mL. Subsequently, around 48 h after the estrogen’s peak, a sudden LH surge occurs, which promotes ovulation 20–26 h later [2,3,4,5,6].

Artificial insemination is an important reproductive technology that focuses on increasing the genetic quality of a population. Hormonal treatments for estrus synchronization are effective, even in anestrous animals [4], and they are required for successful artificial insemination (AI) results, with progestogen-based treatments being the most frequently used. The European Union has imposed strict controls on the use of hormones in animal therapy [7] and, in the case of progestogens, it is prohibited to consume products from animals under treatment, and the withholding period for meat is two days. The progestogens (including natural progesterone) are available as impregnated sponges or silicone devices which are placed intravaginally. In the case of goats, intravaginal sponges containing fluorogestone acetate (FGA) or medroxiprogesterone acetate (MGA) for 11–12 days are the most usual synchronization treatments. There are standard timings for the treatment duration and AI across a range of goat breeds, something that contrasts with the vast differences that have been reported between the breeds of other species, in terms of their productive characteristics or location, among other factors.

The Payoya goat breed is an autochthonous, endangered breed reared in Southern Spain. They were formed by crossbreeding with the Alpine and Pyrenean trunk, and they have high rusticity and a good dairy aptitude, being usually in semi-extensive systems. It is reported that this breed does not show a rigid seasonal reproductive pattern [8]. Autochthonous breeds, as in the case of the Payoya breed, are treated for estrus synchronization using the standard protocols, which might not be the most appropriate for this breed. The absence of physiological knowledge about local goat breeds, which affects their hormonal events [9], could thus reduce the success of reproductive programs.

In short, the present study aims to describe the hormonal periovulatory variations of LH, FSH, progesterone and estradiol in the Payoya goat breed when they are treated for estrus induction and artificial insemination, comparing the results obtained in terms of the animals’ reproductive success.

## 2. Materials and Methods

### 2.1. Animals

This observational study involved a total of 24 multiparous, lactating Payoya-breed goats, with an average weight of 53.0 ± 2.0 kg, aged between 3 and 5 years old, and located in Southern Spain (El Bosque, Cádiz; latitude 36°45′27″ N, longitude 5°30′25″ W). This trial was conducted during April (the seasonal anoestrus), and the last parturition occurs during September–October in the previous year. Only the animals showing a good corporal condition (equal to or higher than 2.5 on a scale from 1.0 to 5.0) and no evident diseases (i.e., absent of lameness, cough, dyspnea, nasal secretion, hyperthermia, mastitis or another illness signs) were selected. The feeding was based on a concentrate, in accordance with the NCR (National Research Council) requirements, and *ad libitum* water access was provided.

This experiment was carried out in accordance with the guidelines set out in the European Union regulations (2010/63) and transposed into Spanish legislation (RD 53/2013). The Ethics Committee on Animal Experimentation of the Andalusia Regional Government approved this study.

### 2.2. Semen Collection and Preparation

The semen was obtained from bucks registered with the National Association of Payoya Breed Farmers (ACAPA). The animals were housed in individual stalls with a concrete floor, large windows and access to an outdoor loafing area at the IFAPA Andalusian Research Center (Hinojosa del Duque, Córdoba; 38.30° N, 5.09° W). The bucks were fed daily with a commercial concentrate (0.5 kg) and given *ad libitum* access to alfalfa hay, water and mineral supplementation blocks. The males were kept in a light-controlled environment where they were exposed to alternating conditions of 2 months of long days and 2 months of short days. To achieve this photoperiod regime, an artificial light at ~200 lx was turned on at 8:00 a.m and switched off at 4:00 p.m. or 12:00 p.m. during the short and long photoperiod treatments, respectively.

In order to freeze the sperm, semen was collected twice a week using an artificial vagina during the favorable breeding season, mainly from September to November. After collection, the volume, sperm motility and concentration were determined. Then, a skimmed milk-based extender was used to dilute the sperm samples and to carry out a washing procedure (centrifugation for 10 min at 700 g). Finally, the semen was diluted with the skim milk extender, containing 7% glycerol, to reach a final concentration of 400 × 10^6^ spermatozoa/mL. The inseminating doses consisted of 0.25 mL straws containing 100 × 10^6^ spz and they were frozen in a programmable biofreezer (Kryo 10–16 II, Planer TM, Sunbury-on-Thames, UK), at −25 °C/min up to −150 °C. Later, the straws were kept in liquid-nitrogen containers. The thawing was carried out in a water bath at 37.5 C for 30 s. The frozen–thawed semen was used for AI only when it complied with the following criteria: a total motility > 45% (ISAS Pro2, Proiser, Valencia, Spain) and membrane functionality > 40% (hypo-osmotic swelling test) [8].

### 2.3. Estrus Induction Treatment and Artificial Insemination

In order to induce estrus in goats, sponges impregnated with fluorogestone acetate (FGA, 20 mg, Chronogest^®^ MSD, Animal Health, Madrid, Spain) were inserted intravaginally (this was considered as day 0). On day 10, 50 µg of cloprostenol (Estrumate, MSD, Animal Health, Madrid, Spain) and 400 IU eCG (Foligon, MSD, Animal Health, Madrid, Spain) were administered intramuscularly. The sponges were withdrawn on day 12. Despite the treatment being administered during the season considered as non-breeding, the goats sometimes show cyclic activity at this time, perhaps due to the latitude; this is the reason that the cloprostenol was included in this protocol.

**Figure 1:**
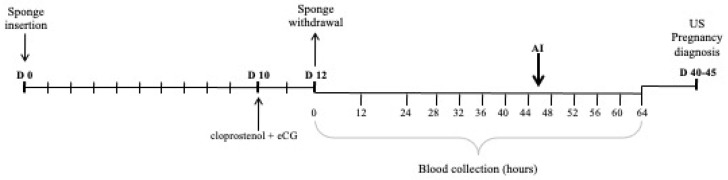
Scheme representing the blood sampling during the experiment.

Subsequently, the goats were maintained in a stall, and blood was collected from their jugular veins as follows: h 0 was considered to be the moment of sponge withdrawal, and blood samples were collected at h 0, h 12, h 24 and then every 4 h, until h 64 (Figure 1). A total of 13 blood samples per animal was obtained, and they were centrifuged at 1000× *g* for 10 min. The plasma was maintained at a cool temperature and stored at −80 °C until analysis.

The fixed-time artificial insemination was carried out by qualified technicians at 46 ± 0.5 h after the sponge withdrawal. The goats were kept standing in a restrained position so that they could not crouch or move about while the catheter was inserted [8]. A frozen sperm straw with 0.25 mL and 100 × 10^6^ sperm was thawed at 37.5 °C for 1 min and loaded into the AI catheter. After the perineal and vulvar area were cleaned, the AI catheter was intravaginally inserted, using a lubricated speculum with a light source to visualize the entry of the cervix, and the semen was deposited through the cervical rings to reach the uterus, when possible.

### 2.4. Pregnancy Diagnosis

Pregnancy was determined around day 40–45 after the AI using an ultrasound scanner (Easy Scan, BCF Technology Ltd., Livingston, Scotland) equipped with a 4.5–8.5 MHz linear array transducer. The probe was placed in the inguinal area and the goats were classified as pregnant when fetuses were visualized.

The conception rate was calculated as the pregnant goats divided by the total inseminated goats × 100. The kidding rate was deemed to be the goats that had a parturition divided by the total number of inseminated goats.

### 2.5. Hormone Assays

The plasma FSH, LH, progesterone and estradiol levels were determined by enzyme-linked immunosorbent assays (Endocrinetech, Endocrine Technologies, Newark, NJ, USA), which had been previously validated for caprine species. The sensitivity levels for the FSH, LH, progesterone and estradiol were 0.5 ng/mL, 0.5 ng/mL, 0.05 ng/mL and 5 pg/mL, respectively. The intra- and interassay variance coefficients for the FSH, LH, progesterone and estradiol were 8.7% and 12.3%, 9.4% and 14.3%, 8.0% and 12.1%, and 8.2% and 9.0%, respectively.

The area under the curve of the LH surge (AUC_LH_) was estimated by summing the values during the defined LH surge induced after the treatment.

### 2.6. Statistical Analysis

The data were analyzed using the IBM SPSS Statistics v. 25 software (Chicago, IL, USA). A descriptive analysis was conducted to identify the maximum hormone levels and the releasing days. The data were analyzed by ANOVA for repeated measures. When *p* < 0.05, the differences were considered as significant. The data are expressed as the mean ± SEM.

## 3. Results

The conception rate after hormonal treatment was 62.5%. A total of 15 goats became pregnant, and the other 9 goats were diagnosed as non-pregnant. The kidding rate was 50% (12/24), which means that the abortion rate was 20% and the mean prolificity was calculated as 2.0 ± 0.2.

The progesterone levels were below 0.5 ng/mL in all the samples, confirming that no functional CLs were present in the ovaries between the end of the induction treatment and 64 h later (i.e., during the sampling period).

### 3.1. LH Level

**Figure 2:**
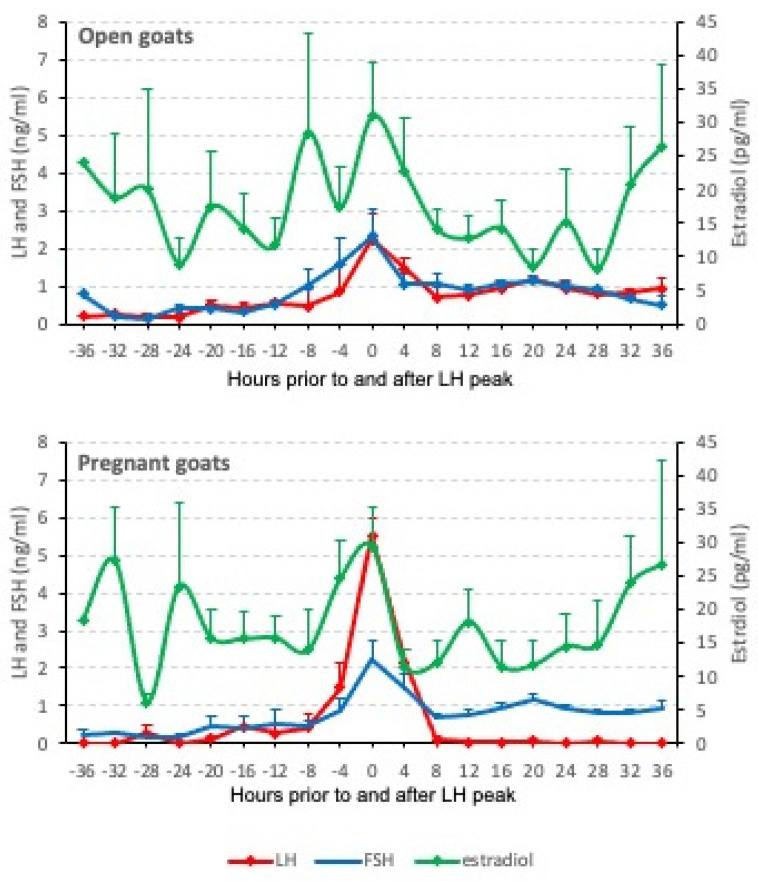
Hormone profiles for LH, FSH and estradiol adjusted to the moment of LH peak in pregnant and open goats. Mean and standard error are shown.

The artificial insemination was carried out 46 h after the sponge withdrawal, and 95.8% of the treated goats showed a preovulatory peak of LH. The LH values ranged between 0 and 8.9 ng/mL. The overall peak of LH was 4.8 ± 0.4, and significantly higher values were identified in the pregnant goats (5.5 ± 0.5 ng/mL vs. 2.2 ± 0.6 ng/mL, *p* < 0.05) (Figure 2).

**Figure 3:**
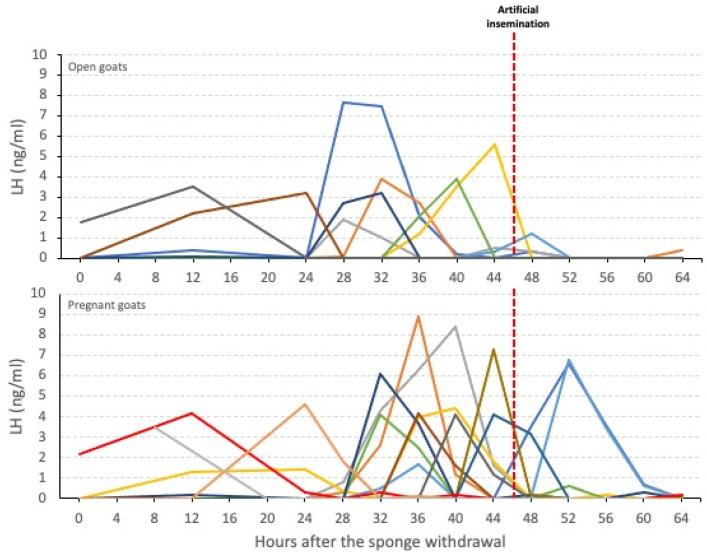
Individual plasma LH profiles in open and pregnant goats after the vaginal sponge withdrawal. Different colorful lines represent individual goats. Red-dashed line indicates the moment of artificial insemination.

The LH peak was detected around 34.96 ± 2.23 h after the sponge removal: in the pregnant goats, it occurred around 36.86 ± 2.8 h; in the non-pregnant goats, the peak was detected slightly earlier, at 32.0 ± 3.7 h after the end of the estrus induction treatment (Table 1). No significant differences were detected for the aforementioned values, and the LH peak times showed a scattered distribution. It was observed that, in the pregnant goats, the LH peak was detected as early as 12 h or as late as 52 h after the end of the treatment (Figure 3).

The overall LH peak duration was 9.91 ± 0.77 h, and no significant differences (*p* > 0.05) were detected between the pregnant and non-pregnant goats (10.2 ± 2.8 h and 9.9 ± 3.4 h, respectively). The majority of the goats showed a LH surge lasting around 8 h, and it was noted that some open goats had a duration shorter than 8 h or longer than 18 h.

Following the estrus induction treatment, the overall area under the LH surge reached 24.89 ± 3.07 ng/mL, with higher values in the pregnant goats (28.09 ± 15.34 ng/mL) than in the open goats (19.90 ± 12.90 ng/mL), although no significant differences were detected.

### 3.2. FSH Levels

**Figure 4:**
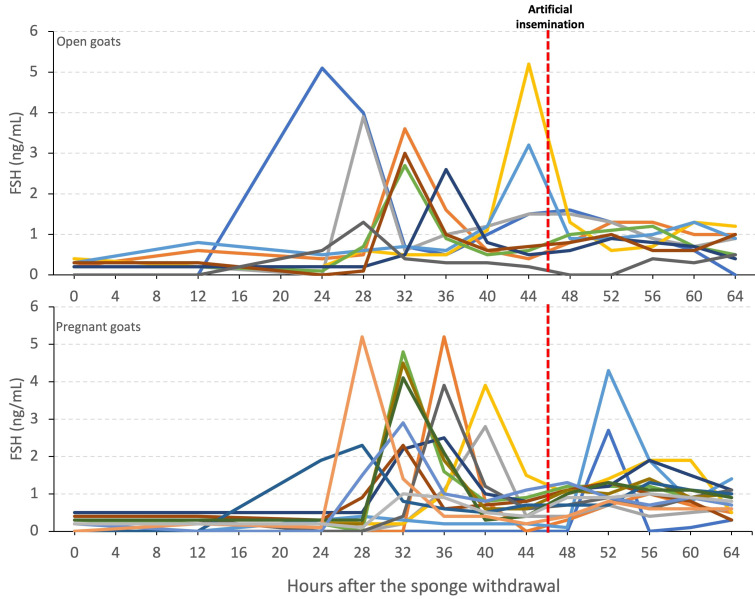
Individual plasma FSH profiles in open and pregnant goats after vaginal sponge withdrawal. Different colorful lines represent individual goats. Red-dashed line indicates the moment of artificial insemination.

The FSH levels ranged between 0 and 5.2 ng/mL, and all the goats showed a single FSH peak around ovulation (Figure 2). The FSH peak was detected at 33.91 ± 0.29 h after the end of the treatment. While the pregnant goats had this peak around 34.86 ± 0.44 h after the treatment (ranging from 28 to 52 h), the peak was slightly earlier in the goats in which pregnancy failed (at 32.44 ± 0.84 h post-treatment, ranging from 24 to 44 h) (Table 1 and Figure 4).

### 3.3. Estradiol Levels

The estradiol showed some rises during the periovulatory period in most of the goats. The higher estradiol peaks were observed around 11.3 ± 3.5 h after treatment in the goats that became pregnant, but it was delayed to 19.0 ± 3.7 h in the case of the non-pregnant goats (Table 1). The estradiol increment was detected from the first sampling (0 h) to 36 h after treatment in both the pregnant and open goat groups.

## 4. Discussion

When estrus induction and synchronization treatments are implemented, it is important to ascertain when they promote the increment of estradiol, and also the timing of the FSH and LH peaks, bearing in mind that the progesterone needs to be at basal levels around ovulation to obtain successful results. The present study, conducted in Payoya goats, demonstrates the different patterns exhibited by pregnant and open goats, with clear differences linked to the LH hormone. It is not easy to record this information, since repeated sampling needs to be carried out, and the difficulty is heightened in endangered and/or autochthonous breeds. The sampling should ideally be conducted every 4 h, which requires great effort, and usually is replaced by an easier methodology, based on the recording of the beginning and the end of estrus signs, although this is less accurate. Ultrasonography has also been used for this purpose, but it is a challenging option for technicians, and the need to restrain the animals may influence the results.

The conception rate obtained in this trial was 62.5%, which is in accordance with the expected results, and in line with the other studies (Table S1) [10,11,12,13,14,15,16,17]. The kidding rate was lower than that reported elsewhere, but the reason for this pregnancy loss remains undetermined.

In Boer x Nubia goats in Mexico [18], the interval between the sponge withdrawal and the onset of estrus behavior has been reported as 17.2 h when eCG was also administered. However, the studies in Ionica goats, using an FGA sponge and PGF2α plus eCG, reported a longer interval (34.7 h) [12]. In the present study, the onset of estrus was not monitored specifically, but it was estimated based on the estradiol increment. In goats treated with FGA for 21 days and 400 IU eCG, other authors have reported that the maximum estradiol levels were reached 18 h after the eCG, showing 44.3 pg/mL, and estrus signs were evident around 22–30 h after the sponge withdrawal [5]. This observation suggests that, after the increment of the estradiol levels, goats exhibit estrus signs around 4–12 h later. Thus, it was concluded that the estrus signs in Payoya goats theoretically appear around 15–34 h after the sponge withdrawal, which is in accordance with the findings of other researchers [12]. One hypothesis is that fertility is reduced after the estrus synchronization treatment if the increment of the estradiol levels is delayed (and thus also the estrus signs).

In the pregnant goats, the estradiol peaks were distributed over 12 h after treatment, while, in the non-pregnant goats, these peaks were scattered throughout a 20-h post-treatment period. Similar findings were reported in Alpine and Saanen goats, synchronized with the progestogens PGF2α and eCG and inseminated 44 h after the sponge withdrawal [19]. The authors in question noted that the goats showing estrus later than 30 h after the end of the treatment exhibited a significant reduction in fertility, in comparison with the goats showing an earlier estrus at 24–30 h. It is possible that a prolonged estradiol release after the synchronization treatment could reduce the fertility in goats. Estradiol is responsible for the induction of estrous behavior and the LH surge and ovulation, and all these events will depend on the follicle growing [20]. A delayed elevation of estradiol after the sponge withdrawal could denote the slower growth of the follicles, preventing the eCG from promoting ovulation in the early-stage follicles and reducing fertility. Thus, it may be that treatments to ensure an earlier estradiol release could increase the pregnancy rate in the Payoya.

The interval from the estradiol rise to the FSH and LH peaks was significantly higher in those Payoya goats that became pregnant after the treatment (23.2 h and 25.3 h for the FSH and LH peaks, respectively) than in those where the pregnancy failed (13.0 and 14.3 h, respectively). However, the interval between the end of the treatment and the FSH and LH peaks did not exhibit significant differences (Table 1). The aforementioned interval to the LH peak was 34.6 h and 32.0 h in the pregnant and non-pregnant goats, respectively, while the interval to the FSH peak was 36.6 h and 33.3 h, respectively. In autochthonous Spanish goats reared for meat, it has been reported that the interval between the end of treatment and the LH surge was 29 h and 36 h in anestrus and the breeding season, respectively [20]. Other studies have reported that the duration of the mentioned interval was 25.7 h, using FGA plus eCG, but significantly longer when other treatments based on prostaglandins or progestogens were used [18]. In Alpine dairy goats treated with MGA plus eCG, this interval was as long as 54.5 h [21]. Similarly, a long interval of around 59.7 ± 2.4 h has been reported when FGA was used in crossbreed dairy goats [22], which means that the LH rise was delayed by around 27 h in comparison with the present study conducted in Payoya goats. The authors concerned observed that this interval was much more delayed (it lasted around 100 h, i.e., 67 h longer than in the present study) when MGA was used for the estrus synchronization. It can be affirmed that the LH peak occurred earlier in the Payoya goats (around 33 h after the end of the treatment) than in other goat breeds and/or treatments, suggesting that it is important to characterize the hormone release during the periovulatory period in order to implement the best estrus induction treatment and obtain satisfactory results after AI. The literature reviewed by the present authors suggests that reproductive success rates are affected not only by the hormonal treatment but also the breed in question.

The duration of the LH surge in the present study was 10.2 ± 2.8 h, which is similar to that reported by the other investigators [18,22]. The ovulation in goats is essentially controlled by the moment at which the LH peak occurs, generally taking place around 20–26 h after the LH peak [3,23]. In the present study, the goats were inseminated 46 h after the sponge withdrawal, and the spermatozoa theoretically reached the oviducts 6–12 h prior to the oocytes being released. It is therefore possible to hypothesize that Payoya goats undergoing estrus FGA-based induction for 12 days, plus PGF2a and eCG, may obtain a good LH peak synchronization, thereby ensuring that the spermatozoa reach the oviduct (and the oocyte) at the appropriate moment. However, it is also hypothesized that an insufficient release of LH is associated with reproductive failure.

The pregnant goats showed higher LH peaks than the open goats. The results show that the goats that became pregnant had, during the LH surge, a LH release of over 14.5 ng/mL, and thus it is possible that the cause of pregnancy failure in three open goats is attributable to the reduced LH release, and this reduced LH release is linked to a short surge duration.

It has been reported that a first FSH peak in crossbreed goats reached 33 ng/mL around the onset of proestrus (day 19 post-estrus), and a second FSH peak reaching 26 ng/mL coincided with the LH peak [24]. However, only one FSH rise was observed in native goats during estrus, highlighting the importance of breed differences. As is the case in the present study of Payoya goats, it was reported that the second FSH peak could be lower or absent when the goats are subjected to estrus synchronization [5].

It is important to highlight that the goats were considered as pregnant around day 45 after the AI. It means that early pregnancy failures could occur, but this study was not designed to detect these. Despite this handicap in the early pregnancy detection, significant variations between both the pregnant and open groups were observed, which could partially explain the pregnancy failures in the goats.

A comprehensive understanding of the chronology of the periovulatory events linked to the induction and synchronization protocols is necessary in order to correctly design such protocols. In this sense, it was affirmed that the knowledge of the occurrence of the LH peak predicts the ovulation, while the estrus occurrence can be less precise and variable [25]. To obtain a successful outcome, it is advisable to carry out the AI around 5 h before ovulation, i.e., around 10–15 h after the LH surge. It has been reported that fertility is reduced when the AI is carried out at less than 5 h after the LH surge [26]. In accordance with this suggestion and based on the LH surge observed in the Payoya goats, AI at 46 h after the sponge withdrawal has a strong case for being deemed correct practice. Reproductive failures after the estrus synchronization treatment could be attributed to LH defects, related to the amplitude, duration and/or timing, in consonance with the studies affirming that fertility is reduced when the LH appears later than 36 h after the sponge removal [27]. A longer interval until the LH surge in goats with a high milk production was observed, suggesting the importance of eCG in these animals [27]. A trial using FGA sponges for 11 days and 300 IU eCG concluded that insemination in anestrous goats in Morocco should be done 53 h after the sponge removal, while, with an increment of eCG (up to 500 IU), AI is recommended at 43 h after the sponge removal [28]. On the other hand, a higher pregnancy rate was observed when the goats were inseminated between 18–24 h after the LH peak [16]; then, in according with the results obtained here, it might be suggested that the delay of the AI until 52 ± 4 h after the sponge withdrawal, instead of 46 h, might improve the pregnancy results when the protocol studied here is used in Payoya goats.

## 5. Conclusions

The present study describes the periovulatory LH, FSH, progesterone and estradiol levels after the estrus induction and synchronization treatment in Payoya goats. Differences were observed in the hormonal pattern in those goats that became pregnant when compared to the non-pregnant individuals. The detected LH peak in the pregnant goats suggests that the insemination was carried out at the correct time, and the reproductive failure after the AI was attributed to the low LH peak level. In short, it is desirable to characterize the hormonal release pattern around ovulation in the different breeds in order to implement the best estrus induction treatment and the optimum time for successful AI, due to the significant variations described in the literature.

## Figures and Tables

**Table 1:** Average intervals (±SEM; h) from the end of the treatment (i.e., sponge withdrawal) to the estradiol rise and LH/FSH peaks in pregnant and open goats. n.s.: no significant differences.

	Interval from Sponge Withdrawal to:								
	Estradiol Rise			LH Peak			FSH Peak		
	Pregnant	Open		Pregnant	Open		Pregnant	Open	
Mean (h)	11.3 ± 3.5	19.0 ± 3.6	n.s.	34.6 ± 3.9	32.0 ± 3.7	n.s.	36.6 ± 2.1	33.3 ± 2.3	n.s.
Min–Max	0–36.0	0–36.0		0–52.0	12.0–48.0		28.0–52.0	24.0–44.0	

## Data Availability

Not applicable.

## Supplementary Materials

The following supporting information can be downloaded at: https://www.mdpi.com/article/10.3390/ani12202853/s1, Table S1: Conception rates in different goat breeds under synchronization treatments and artificial insemination.

## References

[1] Delgadillo J.A., Malpaux B., Chemineau P. (1997). La reproduction des caprins dans les zones tropicales et subtropicales. Prod. Anim..

[2] Bono G., Cairoli F., Tamanini C., Abrate L. (1983). Progesterone, estrogen, LH, FSH and PRL concentrations in plasma during the estrous cycle in goat. Reprod. Nutr. Dev..

[3] Fatet A., Pellicer-Rubio M.T., Leboeuf B. (2011). Reproductive cycle of goats. Anim. Reprod. Sci..

[4] Noakes D.E., Noakes D.E., Parkinson T.J., England G.C.W. (2001). The puerperium and the care of the newborn. Arthur’s Veterinary Reproduction and Obstetrics.

[5] Chemineau P., Gauthier D., Poirier J.C., Saumande J. (1982). Plasma levels of LH, FSH, prolactin, oestradiol-17β and progesterone during natural and induced oestrus in the dairy goat. Theriogenology.

[6] Rawlings N.C., Cook S.J. (1993). LH secretion around the time of the preovulatory gonadotrophin surge in the ewe. Anim. Reprod. Sci..

[7] Boletín Oficial del Estado (2009). Real Decreto 2178/2004, de 8 de Abril, Por El Que Se Modifica el Real Decreto 2178/2004, de 12 de Moviembre, Por El Que Se Prohíbe utilizar Determinadas Sustancias de Efecto Hormonal y Tireostático y Sustancias Beta-Agonistas de uso en la cría de Ganado.

[8] Arrebola F., González O., Torres R., Abecia J.-A. (2014). Artificial insemination in Payoya goats: Factors affecting fertility. Anim. Prod. Sci..

[9] Ramukhithi F.V., Nedambale T.L., Sutherland B., Greyling J.P.C., Lehloenya K.C. (2012). Oestrous synchronisation and pregnancy rate following artificial insemination (AI) in South African indigenous goats. J. Appl. Anim. Res..

[10] Menchaca A., Rubianes E. (2007). Pregnancy rate obtained with short-term protocol for timed artificial insemination in goats. Reprod. Domest. Anim..

[11] Vilariño M., Rubianes E., Menchaca A. (2011). Re-use of intravaginal progesterone devices associated with the Short-term Protocol for timed artificial insemination in goats. Theriogenology.

[12] Martemucci G., D’Alessandro A.G. (2011). Induction/synchronization of oestrus and ovulation in dairy goats with different short term treatments and fixed time intrauterine or exocervical insemination system. Anim. Reprod. Sci..

[13] Arrebola F.A., Pardo B., Sanchez M., López M.D., Pérez-Marín C.C. (2012). Factors influencing the sucess of an artificial insemination program in Florida goats. Span. J. Agric. Res..

[14] Navanukraw C., Khanthusaeng V., Kraisoon A., Uriyapongson S. (2014). Estrous and ovulatory responses following cervical artificial insemination in Thai-native goats given a new or once-used controlled internal drug release with human chorionic gonadotropin. Trop. Anim. Health Prod..

[15] Yotov S., Velislavova D., Dimova L. (2016). Pregnancy rate in Bulgarian White milk goats with natural and synchronized estrus after artificial insemination by frozen semen during breeding season. Asian Pac. J. Reprod..

[16] Llanes A., Whisnant C.S., Knox W.B., Farin C.E. (2019). Assessment of ovulation synchronization protocols in goats and use of pregnancy specific protein B (PSPB) enzyme linked immunoabsorbent assay (ELISA) to determine kid number at birth. Domest. Anim. Endocrinol..

[17] Sun S., Liu S., Luo J., Chen Z., Li C., Loor J.J., Cao Y. (2019). Repeated pregnant mare serum gonadotropin-mediated oestrous synchronization alters gene expression in the ovaries and reduces reproductive performance in dairy goats. Reprod. Domest. Anim..

[18] Rodríguez-Castillo J.C., Pro-Martínez J., Villanueva A., Gallegos-Sánchez J. (2010). Duración del celo y pico preovulatorio de LH en cabras Boer x Nubia sincronizadas con diferentes hormonas en latitud tropical de México. Arch. Latinoam. Anim..

[19] Baril G., Leboeuf B., Saumande J. (1993). Synchronization of estrus in goats: The relationship between time of occurrence of estrus and fertility following artificial insemination. Theriogenology.

[20] Zarazaga L.A., Gatica M.C., Gallego-Calvo L., Celi I., Guzmán J.L. (2014). The timing of oestrus, the preovulatory LH surge and ovulation in Blanca Andaluza goats synchronized by intravaginal progestagen sponge treatment is modified by season but not by body conditions score. Anim. Reprod. Sci..

[21] Alvarez Ramírez L., Ducoing Watty A., Zarco Quintero L., Trujillo García A. (1999). Conducta estral, concentraciones de LH y función lútea en cabras en anestro estacional inducidas a ciclar mediante el contacto con cabras en estro. Vet. Mexico.

[22] Martínez-Álvarez L.E., Hernández-Cerón J., González-Padilla E., Perera-Marín G., Valencia J. (2007). Serum LH peak and ovulation following synchronized estrus in goats. Small Rumin. Res..

[23] Greyling J.P.C., van Niekerk C.H. (1990). Effect of pregnant mare serum gonadotrophin (PMSG) and route of administration after progestagen treatment on oestrus and LH secretion in the Boer goat. Small Rumin. Res..

[24] Leyva-Ocariz H., Munro C., Stabenfeldt G.H. (1995). Serum LH, FSH, estradiol-17β and progesterone profiles of native and crossbred goats in a tropical semiarid zone of Venezuela during the estrous cycle. Anim. Reprod. Sci..

[25] Greyling J., Van Niekerk C. (1990). Ovulation in the Boer goat doe. Small Rumin. Res..

[26] Maurel M.C., Leboeuf B., Baril G., Bernelas D. Determination of the LH peak in dairy goats using an ELISA kit in farm. Proceedings of the 8th Scientific Meeting of the European Embryo Transfer Association.

[27] Leboeuf B., Forgerit Y., Bernelas D., Pougnard J.L., Senty E., Driancourt M.A. (2003). Efficacy of two types of vaginal sponges to control onset of oestrus, time of preovulatory LH peak and kidding rate in goats inseminated with variable numbers of spermatozoa. Theriogenology.

[28] El Kadili S., Raes M., Bister J., Archa B., Kirschvinck N., Chentouf M., Ruiz R., López-Francos A., López Marco L. (2019). Effect of doses of eCG and cloprostenol on oestrus and ovulation induction in North Moroccan goats during anoestrus season. Innovation for Sustainability in Sheep and Goats.

