# Supplementary material for: Periovulatory Hormonal Profiles after Estrus Induction and Conception Rate by Fixed-Time AI in Payoya Goats during the Anestrous Season
Source: Animals (Basel). 2022 Oct 20;12(20):2853. doi: 10.3390/ani12202853 (PMC9597795; doi:10.3390/ani12202853)
Supplement: Supplementary file 1 [file animals-12-02853-s001.zip › animals-1935350-supplementary.pdf]

animals

MDPI

Supplementary Table S1. *Conception rates in different goat breeds under synchronization treatments and artificial insemination.*

Breed	Treatment	Conception rate (%)[a]	References
Alpine	MGA (60 mg) during 5-6 d. + eCG (250 IU) + Delprostenate (160 µg) i.m.,	63,7	[10]
Alpine x Saanen	CIDR during 5 d. and PGFα (15 mg) + eCG (300 IU) at withdrawal	75,3	[11]
Ionic	FGA (45 mg) during 9 d. + cloprostenol (50 µg) on d7 + eCG (400 IU) at withdrawal	41,6 (AI)<br>65,2 (Laparoscopy)	[12]
Florida	FGA (20/45 mg) during 11 d. and cloprostenol (50 µg) + eCG (400 IU) at withdrawal	48,7	[13]
Native Thai	CIDR (re-used) during 14 d, hCG (300 IU) at withdrawal	28,6	[14]
White Bulgarian	FGA (30 mg) during 12 d., eCG (500 IU) at withdrawal	60	[15]
Crossed Boer	CIDR during 6 d., PGFα (15 mg) at withdrawal	64	[16]
	CIDR during 10 d., PGFα (15 mg) at withdrawal	76	
	CIDR during 15 d., PGFα (15 mg) at withdrawal	56	
Xinong Saanen	CIDR during 10 d. + eCG (300 IU) at withdrawal	68,2	[17]

[a] Conception rate: pregnant goats / total inseminated goats
